# Ten Years On – A Survey of Orthoptic Stroke Services in the UK and Ireland

**DOI:** 10.22599/bioj.135

**Published:** 2019-05-08

**Authors:** Lauren Hepworth, Fiona Rowe

**Affiliations:** 1 Department of Health Services Research, University of Liverpool, UK

**Keywords:** orthoptics, stroke, services, provision, vision assessment

## Abstract

**Aim:**

In 2007 a national orthoptic survey identified poor provision of vision assessment for stroke survivors. The purpose of this study is to report a 10-year update of this survey to identify changes in clinical practice over recent years.

**Methods:**

An online practice survey of registered orthoptists (British and Irish Orthoptic Society, BIOS) was undertaken to scope vision services for stroke survivors.

**Results:**

At the time of this survey, there were 223 orthoptic departments and 227 stroke units in the UK and Ireland. 317 responses were received representing 178 orthoptic departments – an 80% response rate for orthoptic departments. Of the respondents, 92% reported having a stroke unit in their hospital. A stroke/vision service was provided by 98% of responding orthoptic departments for 77% of stroke units but with only half providing a vision service on the stroke unit. Only 33% of vision services were funded and funding remains the primary barrier to providing a stroke/vision service. About 85% of respondents were aware of the national clinical guidelines for stroke and the BIOS extended practice guidelines for stroke.

**Conclusions:**

There has been a positive increase in awareness of stroke-related visual impairment and a steady improvement in provision of eye care for stroke survivors. However, there remains a lack of provision of specialist vision services specifically on stroke units which infers a health inequality for stroke survivors who have visual impairment. Their visual impairments can remain undetected and thus undiagnosed and unmanaged due to unsatisfactory patient care.

## Introduction

In 2007, a UK orthoptic profession survey was undertaken with the purpose being to determine orthoptic involvement in stroke services and the type of provision of vision assessment (Rowe 2010). Results of this survey were that 45% of responding services did not provide formal vision assessment for stroke survivors. Of those providing vision assessments, 15% were basic qualitative assessments, primarily of visual fields and ocular motility. At a similar time, a survey was undertaken of current practice by Scottish occupational therapists in which only 9% reported use of a protocol for vision assessments for stroke survivors, and assessments were primarily aimed at visual inattention and hemianopic visual field defects (Pollock et al. 2011).

Since the publication of these surveys a number of national publications have been released or updated which document national clinical guidelines and recommendations. The National Stroke Strategy was published in December 2007 and recognised vision and visual perceptual difficulties as components of multi-faceted stroke specialist rehabilitation and support (Department of Health 2007). The National Clinical Guidelines for Stroke were published in 2008 and subsequently updated in 2012 and 2016 (Intercollegiate Stroke Working Party 2016). These guidelines now recommend orthoptists as part of the core acute stroke unit multi-disciplinary team with specialist assessment and management indicated for visual function deficits (inclusive of visual acuity, eye movements, visual field loss, visual inattention and visual perceptual difficulties). The NICE stroke rehabilitation guidelines published in 2013 advocate screening of stroke survivors for visual difficulties with referral specifically for those with double vision, management for visual field loss and driving advice for those with visual problems (National Institute for for Health and Clinical Excellence 2013).

Currently 1.2 million stroke survivors are living in the UK (Stroke Association 2018). About 60% of stroke survivors will develop new visual problems following their stroke (Rowe et al. 2019) – a sizeable number of stroke survivors living with various types and severity of visual difficulty and causing significant impact to daily life (Hepworth and Rowe 2016). In view of the rise in public awareness campaigns (e.g. Act FAST) for stroke and the changes in national professional and clinical guidelines, the purpose of this study was to revisit UK and Ireland orthoptic services to document changes in the provision of specialised orthoptic care for stroke survivors over the past 10 years since the first 2007 national orthoptic stroke survey.

## Materials and methods

In conjunction with the British and Irish Society (BIOS) an email advert was distributed to all orthoptists registered with their professional body, requesting their participation in a national professional survey of stroke service provision. There are approximately 1440 HCPC registered orthoptists in the UK (Health and Care Professions Council 2018), not all of which will be registered members of BIOS. At the time of the survey, the number of orthoptic departments in the UK and Ireland was 223 (British and Irish Orthoptic Society 2018) and the number of stroke units was 227 (Royal College of Physicians 2017).

**Figure 1:**
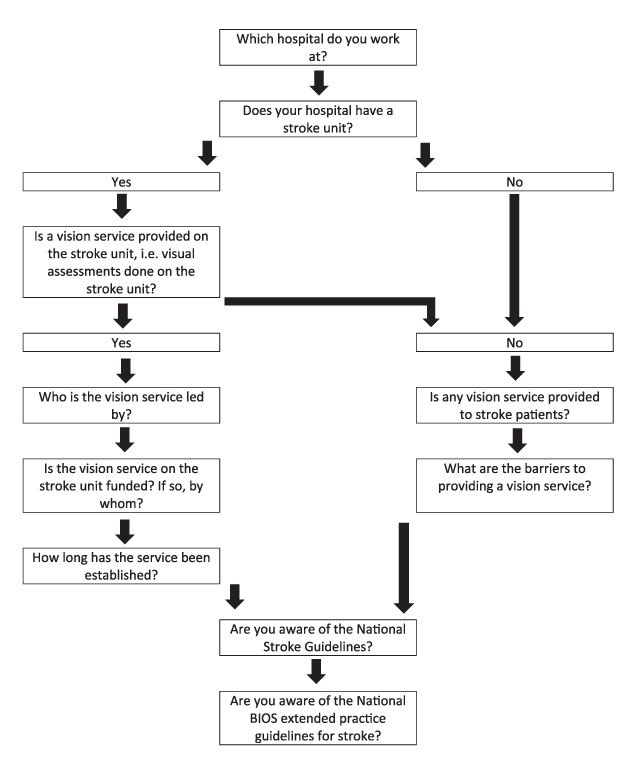
Outline of survey including logic routing.

Specifically the survey asked the questions outlined in Figure 1 and was delivered via a Survey Monkey™ (www.surveymonkey.com) platform. The survey remained open for eight weeks with two reminders emailed out to registered orthoptists by BIOS.

The information provided on the returned survey responses was inputted to a database (SPSS version 24: IBM USA) and descriptive analysis undertaken to combine responses in relation to each of the questions. This study conformed to the Tenets of Helsinki. Institution academic ethical approval was not sought for this study. However, professional organisation approval was obtained. The opening page of the survey included information about the purpose and content of the survey. We deemed that implied informed consent was obtained from those who completed the survey.

## Results

### Hospital responses

**Figure 2:**
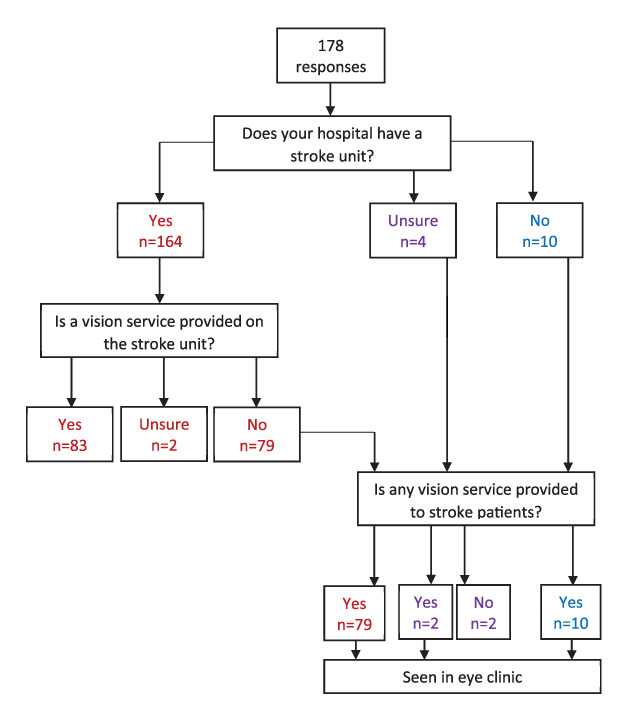
Flow chart of responses.

Three hundred and seventeen responses were received. Duplicate responses from the same department were amalgamated to create one response from 178 orthoptic departments excluding children’s hospitals. This represents a response rate of 79.8% against the number of known orthoptic departments in the UK and Ireland (n = 223). A flow chart of responses is outlined in Figure 2.

The majority (92.1%, n = 164/178) of orthoptic department responses reported having a stroke unit within their Trust.

### Provision of vision services

Four respondents reported not knowing if their orthoptic department provided a stroke vision service, of which three did not answer any further questions. Two of these respondents knew of the stroke unit in their hospital and two were unsure. The provision of a vision service routinely on the stroke unit was reported by approximately half of department responses (47.8%, n = 85/178). This represents a stroke unit orthoptic service of 37.4% (85/227 UK and Ireland stroke units).

Of the 93 department responses that did not provide a vision service on the stroke unit, the majority (45.5%, n = 81/178 departments: 35.7%, n = 81/227 stroke units) provided vision assessments for stroke survivors in the eye clinic. An additional seven of these department responses reported if the patient was unable to come down to the eye clinic, the orthoptist would offer to visit the stroke unit on an ad hoc, non-routine basis. Overall, an orthoptic stroke service (whether provided as in-patient on the stroke unit, or out-patient in the eye clinic) was offered by 174 orthoptic departments (97.8%: 76.7% of stroke units).

**Figure 3:**
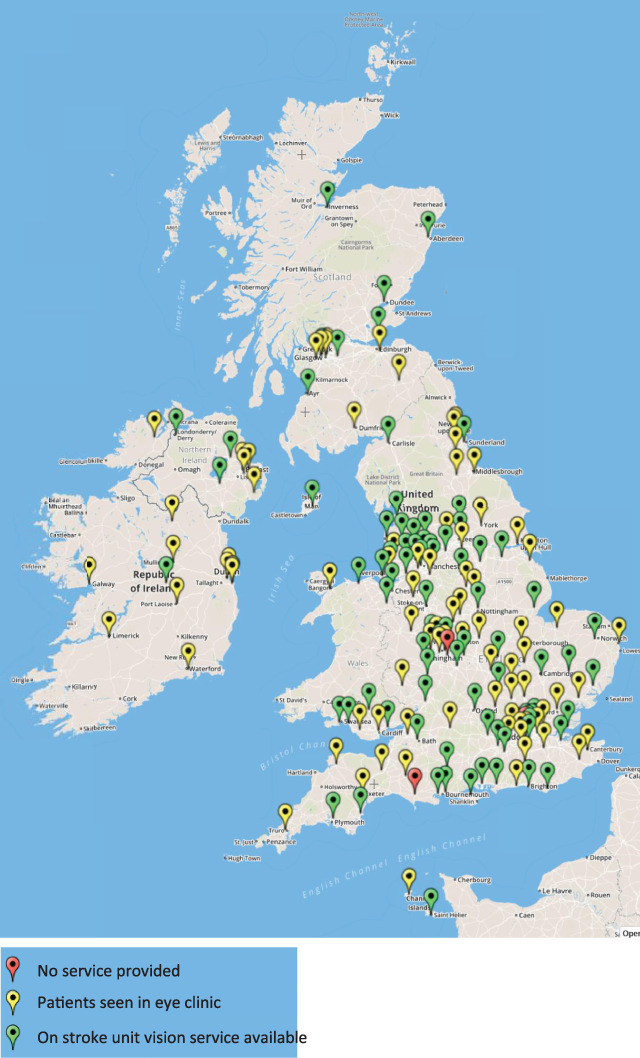
Map of the provision of vision services for stroke across the British Isles. Google. Create Maps: Scribble Maps. 2016 [Available from: www.scribblemaps.com/create#lat=36.879620605027014&lng=-40.78125&z=3&t=hybrid].

The professional group leading the stroke vision service was reported as 77.1% (n = 64) by orthoptists and 16.9% (n = 14) by occupational therapists. A map of the provision of vision services for stroke is outlined in Figure 3.

### Support for vision services

**Figure 4:**
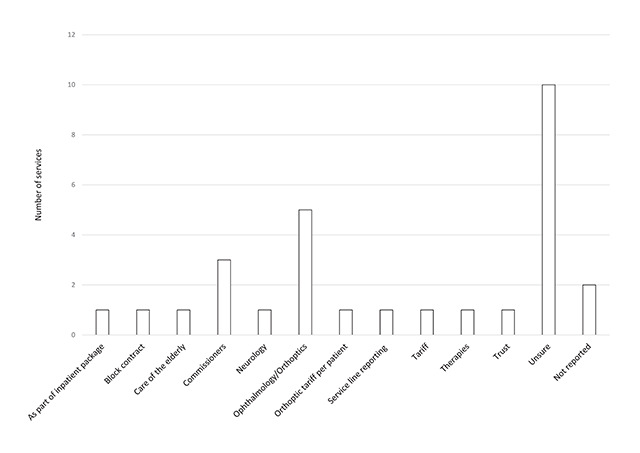
Sources of funding for stroke unit vision services as describes by respondents (n = 28).

**Figure 5:**
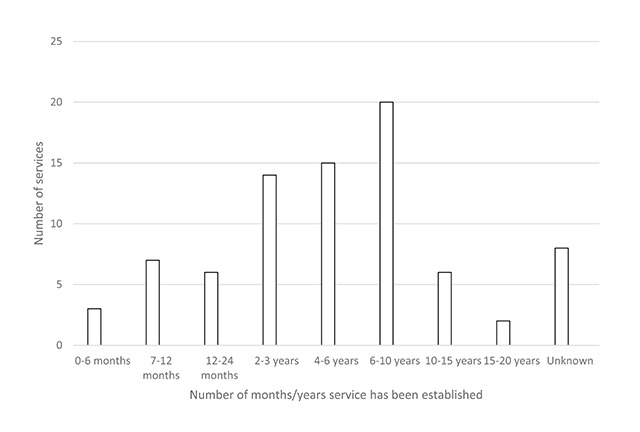
Length of time vision service on the stroke unit has been established (n = 81).

Of those that provide a vision service on the stroke unit, 28 respondents (32.9%) reported this service to be funded. A wide variety of responses were received in reference to who funded the service, which are outlined in Figure 4. These included ten (35.7%) ‘unknown’ responses. Sources of funding included; ophthalmology/orthoptics, orthoptic tariff per patient, commissioners and other hospital departments. These services have been in place for a mean of 69.4 months (5.8 years), SD 58.2 months (4.8 years) and a median of 48 months (4.0 years), range of 2 months to 240 months (20 years), which are outlined in Figure 5.

One primary barrier of funding was identified in providing a vision service on the stroke unit by a high proportion of respondents; no funding from stroke services (n = 49, 57.6%) and no funding from ophthalmic services (n = 37, 43.5%).

### National guideline awareness

The majority of respondents were aware of the national stroke guidelines (n = 162, 85.7%) and the national BIOS extended practice guidelines for stroke (n = 160, 84.7%).

## Discussion

The response rate of this survey was 79.8% of orthoptic departments in the UK. This is considerably greater than the response rate of 42% reported in the first orthoptic stroke survey in 2007 (Rowe 2010). Most departments (93.3%) reported having a stroke unit within their Trust and all provided vision assessments for stroke survivors. However only 45.5% offered their vision service routinely on the stroke unit with more providing vision assessments as an outpatient service in the eye clinic. These figures are considerably better than the 2007 orthoptic survey in which there were no formal vision assessments provided for stroke survivors in 45% of stroke services. This increase in service potentially reflects an increased awareness of stroke-related visual impairment. Indeed, the number of responses indicating awareness of national guidelines increased from 62% (2007 survey awareness of National Service Framework for Older Persons [Stroke]) (Rowe 2010) to 85.7% (current survey awareness of National Clinical Guidelines for Stroke).

However, there is still a sizeable shortfall of orthoptic stroke services provided specifically on the stroke unit. The National Clinical Guidelines for Stroke recommend orthoptists as a core stroke team member and all stroke survivors should be offered vision screening (Intercollegiate Stroke Working Party 2016). This does not happen in some stroke units, representing a health inequality for stroke survivors (Hanna and Rowe 2017). There is a high risk that stroke survivors without obvious signs of visual impairment never have their visual impairment detected and thus live with undiagnosed visual impairment and the consequences of this. These individuals are at greater risk of impaired mobility, falls and accidents, and some individuals inevitably return to driving despite not meeting national driving vision requirements (Pollock et al. 2011). Having orthoptists conduct vision assessments on stroke units represents best practice in order to accurately screen and diagnose presence of visual impairment (Rowe et al. 2016). We support the national recommendation of integration of orthoptic stroke services consistently across the UK to achieve parity of specialist services for the benefit of stroke survivors.

Our survey showed less than one third of orthoptic stroke services were funded specifically and funding was from a variety of sources. Most services had been established for over 4 years. Funding has been established as a major barrier to provision of orthoptic stroke services along with lack of training and knowledge and insufficient management support (Rowe et al. 2015). Funding remained the primary barrier for provision of orthoptic services in this survey. It is of considerable concern that services do not attract funding where national recommendations are to provide these services. Furthermore, there is a clear need as evidenced by published data on numbers of stroke survivors living in the UK (1.2 million) (Stroke Association 2018) with a reported incidence of 60% visual impairment for stroke survivors (Rowe et al. 2019).

There are a number of limitations to this study. Responses were not received from all orthoptic departments so there is the potential that some stroke units from those hospitals have an agreed vision service. Hence our results may underestimate orthoptic stroke service provision. Ward visits for stroke survivors who were unable to come down to eye clinic was not offered as a standard response option within the survey; therefore the number of services offering this could potentially be higher.

There was no formal cost burden analysis as part of this survey. However, some basic estimates can be suggested. For example, the cost of one hip fracture is approximately £25,000 to the NHS (National Institute for for Health and Clinical Excellence 2017). Visual impairment is a widely recognised contributory factor in the cause of falls and hip fractures. Average estimates of orthoptic service cover have been suggested as under £10,000 per annum per hospital (Rowe et al. 2015). We argue that there are cost savings to be made in the NHS through provision of orthoptic services on acute stroke units. Identification of visual impairment triggers the appropriate comprehensive specialist vision assessment, early vision interventions and sharing of information on vision status with stroke survivors, families, carers, medical and therapy teams with sign-posting to further NHS and social care support services.

Advantages of providing orthoptic stroke services are that specialist assessments can be made on the stroke unit which aids the avoidance of misdiagnosis by inaccurate vision screening by non-eye trained staff on the stroke unit, avoids the need for in-patient cross-department referrals with related waiting times, prevents depletion of ward staff when the patient is accompanied to the out-patient eye department, provides immediate feedback to the stroke team so that visual impairment information can be used to inform any adjustments to therapy programmes/plans for the individual patient, avoids unwarranted return to driving, access to immediate visual impairment treatment and advice, and access to advanced treatment such as prisms for double vision.

## Conclusions

We report a 10-year update survey on provision of orthoptic services for stroke survivors. A clear increase in provision of these services has occurred with greater reporting of awareness of issues due to stroke-related visual impairment. However, there remain areas with no provision of vision care for acute stroke survivors which represents a health inequality for these people. Funding remains the primary barrier to the provision of these services despite consistent recommendations from national stroke and vision guidelines for the provision of vision services on acute stroke units with specialist assessment of central and peripheral vision, eye movements and visual perception with their timely management.
